# The Effectiveness of Rehabilitation Interventions for Improving Leisure Participation Following Stroke: Protocol for a Systematic Review

**DOI:** 10.2196/71353

**Published:** 2026-02-26

**Authors:** Serena Alves-Stein, Natasha A Lannin, Kylie Wales, Sharon Kramer, Laura Jolliffe

**Affiliations:** 1 Department of Neuroscience, School of Translational Medicine, Monash University, Level 6, 99 Commercial Rd, Melbourne, VIC 3004, Australia; 2 Department of Occupational Therapy, Alfred Care Group, Bayside Health, Level 6, 99 Commercial Rd, Melbourne, 3004, Australia, +61 3 9903 0555; 3 Faculty of Health Sciences, School of Allied Health, Australian Catholic University, North Sydney, Australia; 4 MedTechVic Hub, Swinburne University, Hawthorn, Australia; 5 Department of Occupational Therapy, School of Primary and Allied Health Care, Monash University, Australia; 6 Department of Occupational Therapy, Peninsula University Hospital, Peninsula Care Group, Bayside Health, Australia; 7 National Centre for Healthy Ageing, Melbourne, Australia

**Keywords:** stroke, leisure activities, rehabilitation, community integration, occupational therapy, stroke rehabilitation, social engagement

## Abstract

**Background:**

Leisure participation is an important rehabilitation goal for survivors of stroke. Following stroke, there is a reduction in leisure participation; however, the focus of rehabilitation is typically on remediation of personal care activities and mobility. Furthermore, previous systematic reviews and current clinical practice guidelines provide inconsistent recommendations for rehabilitation interventions to improve leisure participation. This highlights the need for a comprehensive and targeted review of the literature to help inform clinical practice.

**Objective:**

We propose a systematic review to synthesize data on the effectiveness of rehabilitation interventions to increase leisure participation in adult survivors of stroke, taking into account time since stroke and intervention context.

**Methods:**

Searches will be conducted in MEDLINE, CINAHL, Embase, and CENTRAL. We will include randomized controlled trials and nonrandomized controlled trials that include adult survivors of stroke and test the effectiveness of rehabilitation interventions for leisure participation. Eligible interventions will be those that aim to improve leisure participation or where leisure participation is an outcome of interest. Two reviewers will independently screen full-text articles, and one reviewer will extract data, with a second reviewer providing confirmation. The Physiotherapy Evidence Database Scale will be used to assess the methodological quality of the studies. A random-effects meta-analysis will be performed, and a Cochran *Q* test will assess heterogeneity among studies. Outcome measures of leisure participation may include measures of amount, satisfaction and confidence, and performance. Secondary outcomes will include quality of life measures, adverse events, and resource use.

**Results:**

Results will be discussed based on subgroup analyses where possible, including (1) time since stroke (early subacute, late subacute, and chronic), (2) delivery of the intervention (group or individualized), and (3) type of intervention (functional impairment, leisure education, and recreation participation). At the time of this protocol publication, the systematic review has progressed to data analysis, with publication of results expected in early 2026.

**Conclusions:**

The findings of this review will increase understanding of effective rehabilitation practices to increase leisure participation after stroke and may contribute to updates of existing clinical practice guidelines for stroke rehabilitation.

## Introduction

Stroke is a leading cause of disability worldwide, with data suggesting that the increase in incidence in younger age groups has led to an increase in years lived with disability [1,2]. Stroke-related impairments, such as motor, cognitive, and communication impairments, impact activity participation [3]. Typically, rehabilitation programs tend to focus on remediating impairments and improving independence in basic activities of daily living such as feeding and bathing [4,5], all seen as key to supporting discharge from hospital to home [6]. However, it is important that social and community-related needs and goals are also addressed during stroke recovery [5]. With an increased number of survivors of stroke returning to living in the community, there is an increasing need for community-based rehabilitation programs to enable community engagement [7], which includes leisure participation.

Worldwide, survivors of stroke participate less in leisure activities [8-11]. Furthermore, there is a change in the type of leisure activities they engage in, with an increase in sedentary, home-based activities such as watching television [4,7]. Engaging in leisure following stroke has benefits for adjustment, resilience [12], and quality of life [11]. Leisure participation has been found to promote positive mental health outcomes, reduce the risk of cognitive decline, and reduce the risk of physical disability and disease related to aging [13-16].

Despite the importance of leisure participation, clinical practice guidelines provide limited direction on how to address reduced leisure participation. The evidence underpinning guideline recommendations is considered weak [17]. This is likely due to a lack of research and variability in study findings. For example, the Australian guidelines recommend targeted occupational therapy programs to address leisure participation [17], whereas the American guidelines recommend education and support to overcome barriers to physical activity [18]. In comparing the guideline recommendations, it is also notable that some of the cited studies are specific to leisure interventions [19-23], whereas others test the effect of non–leisure-specific interventions on broader participation outcomes [24-26].

A more comprehensive and targeted search of the literature can provide greater clarity on clinical recommendations for poststroke leisure rehabilitation. There is a need to better understand not only the effectiveness of leisure interventions but also the context in which these interventions are delivered. This includes considering time since stroke, the intervention delivery format (eg, one-on-one or in a group), and the content of leisure interventions.

Given these needs, this systematic review will synthesize data from leisure intervention studies that measure leisure participation for community-dwelling survivors of stroke. The review aims to address the following research question: what is the effectiveness of rehabilitation interventions in increasing leisure participation in adult survivors of stroke, taking into account time since stroke and intervention context?

## Methods

### Ethical Considerations

This systematic review will analyze publicly available data from previously published peer-reviewed articles and will not recruit new human participants. Therefore, approval from a research ethics committee is not required.

### Study Design

This systematic review protocol has been developed according to the PRISMA-P (Preferred Reporting Items for Systematic Reviews and Meta-Analyses–Protocols) statement [27]; the PRISMA (Preferred Reporting Items for Systematic Reviews and Meta-Analyses) statement will guide the systematic review reporting. The protocol has been registered in PROSPERO (CRD42024547133).

### Eligibility Criteria

#### Overview

The primary objective of this review is to synthesize effectiveness research on the use of leisure rehabilitation interventions after stroke. We will include publications from any date in our search. We acknowledge that this may influence key definitions of “leisure” used in the context of research [28-30]. Challenges noted to date arise from whether leisure must always be about how one uses discretionary time as opposed to the view that an activity may be considered leisure provided there is a pleasurable mental state that occurs during the activity [28]. Cultural context may also influence whether an activity would be considered leisure [30], and we acknowledge that this may shift over time. Consistent with definitions reported in publications over the past 2 decades, we will consider leisure activities those activities that are not necessary or obligatory to survival or to necessities of life, are activities of choice, and are done for the purpose of enjoyment or pleasure [28,30].

#### Types of Studies

Studies will be included if they are full publications of pretest-posttest randomized or nonrandomized controlled trials that assess the effects of the interventions (including those with waitlist control groups).

#### Population and Setting

Studies that include adults (aged ≥18 years) diagnosed with stroke who are community dwelling will be included. Studies involving mixed diagnoses will be included in cases in which the data from those with a diagnosis of stroke can be extracted or where ≥75% of the participants have a diagnosis of stroke. Studies involving mixed age groups will be included in cases in which the data from those aged ≥18 years can be extracted or where ≥75% of the participants are aged ≥18 years. Studies will be excluded if they relate to children and young people aged <18 years (including those with childhood-acquired stroke) or include participants who reside in a care-based facility (eg, residential aged care or group home programs).

#### Intervention

Studies that provide an intervention aimed at improving leisure participation will be included. The leisure ability model [31] will be used to provide a conceptual framework for describing interventions targeting leisure participation. The leisure ability model describes 3 “categories of service” for providing leisure interventions: functional impairment, leisure education, and recreation participation [31]. Leisure interventions will be provided by rehabilitation professionals, including but not limited to occupational therapists, recreational therapists, physiotherapists, speech and language therapists, nurses, social workers, and allied health assistants. Mode of delivery can include individual or group-based interventions. We will exclude studies in which leisure participation was not a target of intervention for all participants or was not part of the intervention protocol.

#### Comparison Interventions

Included studies may have either an active comparison, such as those in which participants receive usual care or another nonleisure intervention, or an inactive (no-treatment) comparison. Usual care in the included studies refers to rehabilitation that addresses impairment and/or participation goals but not specifically leisure (eg, addressing self-care and walking ability). A vs B–type intervention studies will be included in cases in which there is a comparison of 2 or more interventions where at least 1 addresses leisure participation.

#### Outcomes

To be eligible for inclusion, studies must use a leisure-specific outcome measure. Leisure participation may be assessed as amount, satisfaction or confidence, or performance; where possible, we will analyze each separately. Examples of how these domains could be measured include the following:

- Amount—participant diary or Nottingham Leisure Questionnaire [32]
- Satisfaction or confidence—Leisure Satisfaction Scale [33]
- Performance—participant self-reported performance rating (eg, goal attainment scaling of a leisure performance goal [34])

Additional outcomes that will be reviewed include quality of life, adverse events, and resource use, where reported. Where available, economic evaluation reports will be assessed. We will exclude measures primarily assessing engagement in paid employment, volunteering, or homemaking.

### Search Strategy

Electronic searches will be conducted in MEDLINE, CINAHL, The Cochrane Library, and Embase. Hand searches of the reference lists of included studies and any related systematic reviews will be conducted to identify further potentially eligible studies. The search strategy will include terms related to stroke, rehabilitation, and leisure. Table 1 provides the search terms that will be used to search for and identify relevant studies. The complete search strategy for each database is available in Multimedia Appendix 1.

**Table 1.** Systematic review search terms structured for MEDLINE.^a^

	Population (stroke)	Intervention (rehabilitation)	Outcome (leisure)
Text words	“(Stroke or cerebrovascular accident or cerebr* vasc* or cva* or brain infarct* or cerebral infarct* or brain vascular accident or poststroke or post-stroke or hemipleg* or hemipare*).ti,ab,kw.”	“(rehabilitation or community health services or allied health).ti,ab,kw.”	“(leisure or hobb* or recreation* or recreational activit* or meaningful) adj3 activit*) or community) adj3 engagement) or community) adj3 integration) or leisure) adj3 (activit* or exploration or participation or education or counselling)).ti,ab,kw.”
MeSH^b^ terms	“stroke/ or brain infarction/ or hemorrhagic stroke/ or ischemic stroke/”	“rehabilitation/ or neurological rehabilitation/ or stroke rehabilitation/ or occupational therapy/ or recreation therapy/exp Rehabilitation/”	“exp Leisure Activities/ leisure activities/ or recreation/”

a Search terms and Boolean operators will be tailored for each database.

b MeSH: Medical Subject Headings.

### Procedures

All studies identified via the search strategy will be uploaded to Covidence (Veritas Health Innovation) [35], and duplicates will be removed. One author will independently screen studies for eligibility based on title and abstract, and all excluded articles at the title and abstract stage will be reviewed by a senior researcher to ensure that no articles are inadvertently excluded. Full-text articles will then be independently reviewed by 2 authors to determine eligibility. Disagreements will be resolved first through discussion; however, if consensus is not reached, a third research team member will complete an independent review. Study authors may be contacted to clarify details and/or data. The results of the screening process will be provided in detail using a PRISMA study flowchart.

One reviewer will extract data using a predetermined form; all data extraction will be independently verified by the senior author (LJ). If discrepancies occur, these will be resolved through discussion between the authors. If not resolved, a third author will independently extract the data. The data extraction form will include variables necessary for describing the studies and conducting data analysis, including characteristics of the participants (including age, gender, time since stroke, diagnostic details, baseline leisure participation, and comorbidities), study methods (design, randomization, trial duration, follow-up time points, and information required for risk-of-bias assessment), sample size, country of study, intervention details (according to the TIDieR [Template for Intervention Description and Replication] checklist items [36]), outcomes, and results (number of participants in the analysis, summary data for each intervention group, and estimate of effect along with measure of variance [eg, mean difference and 95% CI]).

The Physiotherapy Evidence Database Scale [37] will be used to examine the risk of bias by one reviewer, and this will then be independently verified by a second reviewer. Any disagreements in appraisal score will be addressed through consensus. A higher score indicates a higher methodological quality of the trial. A score of <4 will indicate poor quality, and these studies will be excluded from meta-analysis. Studies that are excluded from the meta-analysis due to the risk of bias will still be reported descriptively in the text of the review.

### Statistical Analysis

#### Overview

For all outcomes, summary data will be presented by group and reported as estimate of effect size and CI as feasible. If sufficiently homogeneous in terms of clinical (eg, patient characteristics), methodological (eg, study design), and statistical (eg, forest plot consistency) characteristics, statistical analyses will be conducted to determine the treatment effect using a random-effects model. A random-effects model will be used as it is expected that the eligible studies will estimate different intervention effects, contributing to between-study heterogeneity [38]. Statistical heterogeneity will be assessed using the *I*^2^ test and Cochran *Q* test, which is based on a chi-square distribution. If the *I*^2^ is >80%, heterogeneity will be explored through sensitivity analyses, which will include analyses of studies with low or unclear risk of bias (excluding studies with a high risk of bias) and subgroup analyses. Clinical and methodological heterogeneity will be discussed by the reviewer team (ie, interventions, populations, and assessment methods) to inform subgroup analyses. If meta-analysis is not appropriate as a result of substantial heterogeneity (*I*^2^>80%), a narrative synthesis of the findings from the included studies will be provided.

For continuous data, the treatment effect will be calculated using standardized mean differences and 95% CIs in cases in which different studies use different scales to assess the same outcome. Mean differences and 95% CIs will be calculated in cases where studies have used the same measurement tool. For dichotomous outcomes, a relative risk ratio will compare the risk of an event occurring between the study intervention groups. In cases in which studies report more than 1 leisure participation outcome construct, we will use their reported primary outcome in the main analysis. Other reported outcome constructs will be included in subgroup analyses.

#### Subgroup Analyses

As described in the eligibility criteria, leisure participation outcome measurement domains are amount, satisfaction or confidence, and performance. Where possible, these outcomes will be analyzed separately. To investigate intervention context, further subgroup analyses may include time since stroke, delivery of the intervention, and type of intervention where possible. In accordance with the Stroke Recovery and Rehabilitation Roundtable recommendations, poststroke time frames will be classified as early subacute (7 days to 3 months), late subacute (3-6 months), and chronic (>6 months) [39]. Noting the Stroke Recovery and Rehabilitation Roundtable point regarding understanding the efficacy of commencing recovery trials in the early subacute phase, subgroup analysis of time since stroke will compare <3 months (early subacute) and >3 months (late subacute and chronic) after stroke. Subgroup analyses on delivery of the intervention will compare individually provided therapies with group programs. Intervention content will be categorized using the leisure ability model categories of service [31].

## Results

At the time of publication of this protocol, this systematic review has progressed with searches completed in September 2025 and data analysis completed in October 2025. Results will be discussed based on subgroup analyses where possible, including:

1. time since stroke (early subacute, late subacute, and chronic);
2. delivery of the intervention (group or individualized); and
3. type of intervention (functional impairment, leisure education, and recreation participation).

A manuscript for the systematic review results is being finalized and is anticipated to be published in the first half of 2026.

## Discussion

### Overview

The findings of this review will benefit researchers, clinicians, and people living in the community who have experienced stroke. For academic researchers, the review will have the potential to identify gaps in available evidence, thereby highlighting key areas for future research. For clinicians, the findings will inform clinical practice guidelines and, thereby, influence intervention selection. For people living in the community who have experienced stroke, the findings may shed light on how to improve leisure participation in the longer term, something that has to date remained unknown. Specifically, this review aims to provide greater clarity and detail on how to deliver effective leisure rehabilitation by considering the content of the interventions in the included studies and exploring other factors such as when leisure rehabilitation should be provided and what aspects of leisure participation are improved.

### Comparison With Prior Work

Leisure participation following stroke is an important participation goal in stroke rehabilitation; however, systematic reviews to date have not systematically located or comprehensively analyzed the body of research, limiting the ability to draw reliable conclusions. Two previous systematic reviews [19,40] that included the topic of leisure in stroke recovery have methodological limitations influencing the interpretation of their results. Neither review required studies to include a leisure-specific outcome measure, and the 2 reviews used different working definitions of leisure interventions. The studies had a broad scope, which limited their ability to provide a targeted and in-depth analysis of the efficacy of leisure rehabilitation. These limitations have been taken into consideration in the design of this systematic review, which is reflected in the decisions to use subgroup analyses to explore outcome measure constructs, intervention delivery, and intervention content.

### Strengths and Limitations

A key strength of this study is that it addresses the identified limitations of previous systematic reviews by focusing specifically on leisure rehabilitation and leisure-specific outcomes in a stroke population. This allows for a focused analysis and synthesis of results to inform future research and clinical practice. As with all studies, there are potential limitations. By design, our search is limited to studies published in English. We have also used a single reviewer for screening titles and abstracts, which may increase the risk of missed studies, and had a single reviewer complete data extraction, which may result in errors. We have managed these potential errors with verification of these steps by a second author, as described in the Methods section. With regard to statistical methods, we will report *I*^2^ values to quantify heterogeneity, which may not indicate the extent to which true effects vary across different populations or settings. In addition, we anticipate the following challenges. During the search phase, we anticipate that locating and identifying relevant studies may be a challenge if studies include leisure outcomes among other outcomes of interest or there is variation in how leisure interventions are defined or described. We also acknowledge that how leisure is measured may vary across studies in addition to the outcome measures used. Such variability may limit the ability to meta-analyze data from the included studies.

### Conclusions

Summarizing trials completed to date will allow for the analysis of effectiveness alongside discussion of contextual factors that may influence leisure rehabilitation intervention delivery. Thus, this systematic review will help in the decision-making of researchers and clinicians, ultimately advancing the field of rehabilitation.

## Supplementary material

10.2196/71353 Multimedia Appendix 1 Search strategy for each database.

## Abbreviations

PRISMA: Preferred Reporting Items for Systematic Reviews and Meta-Analyses
PRISMA-P: Preferred Reporting Items for Systematic Reviews and Meta-Analyses–Protocols
TIDieR: Template for Intervention Description and Replication
