# Supplementary material for: The Effectiveness of Rehabilitation Interventions for Improving Leisure Participation Following Stroke: Protocol for a Systematic Review
Source: JMIR Res Protoc. 2026 Feb 26;15:e71353. doi: 10.2196/71353 (PMC12945102; doi:10.2196/71353)
Supplement: Multimedia Appendix 1 [file resprot-v15-e71353-s001.docx]

**Supplementary material**

**MEDLINE search strategy**

| **Set** | **Search statement** |
| --- | --- |
| 1. | (Stroke or cerebrovascular accident or cerebr* vasc* or cva* or brain infarct* or cerebral infarct* or brain vascular accident or poststroke or post-stroke or hemipleg* or hemipare*).ti,ab,kw |
| 2. | exp Stroke/ |
| 3. | 1 or 2 |
| 4. | (rehabilitation or community health services or allied health).ti,ab,kw. |
| 5. | exp Rehabilitation/ |
| 6. | 4 or 5 |
| 7. | ((((((((leisure or hobb* or recreation* or recreational activit* or meaningful) adj3 activit*) or community) adj3 engagement) or community) adj3 integration) or leisure) adj3 (activit* or exploration or participation or education or counselling)).ti,ab,kw. |
| 8. | exp Leisure Activities/ |
| 9. | 7 or 8 |
| 10. | 3 and 6 and 9 |

**Embase search strategy**

| **Set** | **Search statement** |
| --- | --- |
| 1. | (Stroke or cerebrovascular accident or cerebr* vasc* or cva* or brain infarct* or cerebral infarct* or brain vascular accident or poststroke or post-stroke or hemipleg* or hemipare*).ti,ab,kw. |
| 2. | exp cerebrovascular accident/ |
| 3. | 1 or 2 |
| 4. | (rehabilitation or community health services or allied health).ti,ab,kw. |
| 5. | exp rehabilitation/ |
| 6. | 4 or 5 |
| 7. | ((((((((leisure or hobb* or recreation* or recreational activit* or meaningful) adj3 activit*) or community) adj3 engagement) or community) adj3 integration) or leisure) adj3 (activit* or exploration or participation or education or counselling)).ti,ab,kw. |
| 8. | leisure/ or recreation/ |
| 9. | 7 or 8 |
| 10. | 3 and 6 and 9 |

**CINAHL search strategy**

| **Search ID#** | **Search Terms** |
| --- | --- |
| S1 | ( Stroke or cerebrovascular accident or cerebr* vasc* or cva* or brain infarct* or cerebral infarct* or brain vascular accident or poststroke or post-stroke or hemipleg* or hemipare* ) OR AB ( Stroke or cerebrovascular accident or cerebr* vasc* or cva* or brain infarct* or cerebral infarct* or brain vascular accident or poststroke or post-stroke or hemipleg* or hemipare* ) OR SU ( Stroke or cerebrovascular accident or cerebr* vasc* or cva* or brain vascular accident or poststroke or post-stroke or hemipleg* or hemipare*) |
| S2 | (MH "Stroke+") |
| S3 | S1 OR S2 |
| S4 | ( rehabilitation or community health services or allied health ) OR AU ( rehabilitation or community health services or allied health ) OR SU ( rehabilitation or community health services or allied health ) |
| S5 | (MH "Rehabilitation+") |
| S6 | S4 OR S5 |
| S7 | ( leisure or hobb* or recreation* or recreational activit* or meaningful) N3 activit*) or community) N3 engagement) or community) N3 integration) or leisure) N3 (activit* or exploration or participation or education or counselling ) OR AB ( leisure or hobb* or recreation* or recreational activit* or meaningful) N3 activit*) or community) N3 engagement) or community) N3 integration) or leisure) N3 (activit* or exploration or participation or education or counselling ) OR SU ( leisure or hobb* or recreation* or recreational activit* or meaningful) N3 activit*) or community) N3 engagement) or community) N3 integration) or leisure) N3 (activit* or exploration or participation or education or counselling ) |
| S8 | (MH "Leisure Activities+") |
| S9 | S7 OR S8 |
| S10 | S3 AND S6 AND S9 |

**Cochrane CENTRAL search strategy**

| **Set** | **Search statement** |
| --- | --- |
| 1. | (Stroke or cerebrovascular accident or cerebr* vasc* or cva* or brain infarct* or cerebral infarct* or brain vascular accident or poststroke or post-stroke or hemipleg* or hemipare*).ti,ab,kw. |
| 2. | exp stroke/ |
| 3. | 1 or 2 |
| 4. | (rehabilitation or community health services or allied health).ti,ab,kw. |
| 5. | exp Rehabilitation/ |
| 6. | 4 or 5 |
| 7. | ((((((((leisure or hobb* or recreation* or recreational activit* or meaningful) adj3 activit*) or community) adj3 engagement) or community) adj3 integration) or leisure) adj3 (activit* or exploration or participation or education or counselling)).ti,ab,kw. |
| 8. | exp leisure activities/ |
| 9. | 7 or 8 |
| 10. | 3 and 6 and 9 |
